# Coronal subluxation of the tibiofemoral joint before and after anterior cruciate ligament reconstruction

**DOI:** 10.1186/s12891-021-04798-1

**Published:** 2021-10-28

**Authors:** Ruibo Li, Xingyue Yuan, Peng Fu, Jianjun Zhang, Yuehong Liu

**Affiliations:** 1 Department of Orthopaedics, Peoples’ Hospital of Deyang City, No. 173, section 3, North Taishan Road, Deyang, 618000 Sichuan Province China; 2 Medical College, Qingdao University, Qingdao, 266000 Shandong Province China

**Keywords:** Anterior cruciate ligament reconstruction, Tibiofemoral joint, Coronal subluxation, Radiograph

## Abstract

**Background:**

Studies have shown that medial subluxation of the tibia occurs after anterior cruciate ligament (ACL) rupture. However, it is unclear whether anterior cruciate ligament reconstruction (ACLR) can correct tibial coronal subluxation.

**Purpose:**

To determine whether the tibia is medially subluxated after ACL rupture, and whether ACLR can correct medial subluxation of the tibia.

**Study design:**

Case series; Level of evidence, 4, Retrospective clinical study.

**Methods:**

The distance of tibial coronal subluxation before and after ACLR surgery was measured in 48 patients with ACL rupture and meniscus injury. Tibiofemoral subluxation was defined as the perpendicular distance between the long axis of the tibia and a second parallel line originating at the most proximal aspect of the femoral intercondylar notch. To determine the long axis of the tibia, two circles separated by 5 cm were centered on the proximal tibia. The proximal circle is 5 cm from the tibial plateau, and the distal circle is 5 cm from the proximal circle. The line passing through the center of the two circles was considered the long axis of the proximal tibia. Care was taken to ensure that each patient lied on the back with their patellae facing upward, to minimize rotational variation among the radiographs. At the same time, 30 patients with simple meniscus injury who underwent arthroscopy during the same period were selected to determine the degree of tibiofemoral coronal subluxation as the baseline value. The changes before and after operation were compared, as well as the differences with the baseline data.

**Result:**

The average follow-up period was 21.2 ± 5.8 months. The average distance of tibial coronal subluxation before ACLR was 5.5 ± 2.1 mm, which was significantly different from that of baseline group (7.3 ± 2.1 mm) (*P* < 0.001). The tibial subluxation after ACLR was 7.7 ± 2.6 mm, which was significantly different from that before operation (*P* < 0.001). There was no significant difference in the distance between postoperative tibial subluxation and baseline group (*P* = 0.472).

**Conclusion:**

The tibia was coronally medially subluxated after ACL rupture. ACLR can correct the medial subluxation of tibia. This finding is helpful in the diagnosis of ACL rupture, and can be used to assess the imaging status of the tibiofemoral joint on the coronal plane during or after ACLR.

## Background

The anterior cruciate ligament (ACL) rupture is a destructive injury that can have a long-term impact on the health of the knee and is usually treated with anterior cruciate ligament reconstruction (ACLR) [1]. ACLR aims to restore knee joint function, stability and biomechanics which are compromised after ACL injury [2, 3], and in this way to prevent the onset of early posttraumatic articular cartilage degeneration [4–6].

The ACL is obliquely connected between the femur and the tibia, indicating that the ACL can resist the coronal load of the tibiofemoral joint and prevent the tibia from shifting medially [7]. Medial Subluxation of the tibia after an ACL rupture has been observed not only on radiographs but also in cadaver studies [8, 9]. The medial subluxation of the tibia inevitably leads to abnormal contact stress between the tibiofemoral joint [10], and may lead to tibial spine impact sign, resulting in complications such as knee cartilage injury and degeneration [11, 12]. Therefore, in the process of ACLR, we should pay attention not only to the sagittal relationship of tibiofemoral joint, but also to the coronal relationship.

Many studies have shown that replicating the angle and size of the original ligament and reconstructing the ACL in the anatomic footprint area can greatly improve knee biomechanics and clinical outcomes [8, 13–15]. However, rotational kinematics is still difficult to fully recover to the initial state [15–18].

Recovery of tibiofemoral articulation is closely related to recovery of knee function after ACLR [19]. However, whether ACLR can correct tibiofemoral coronal plane subluxation remains unclear, as no studies have been conducted on this topic. The aim of this study was to investigate whether ACLR can correct tibiofemoral coronal subluxation by measuring the distance of tibial coronal plane subluxation before and after ACLR in patients with ACL rupture. It was hypothesized that ACLR could correct tibiofemoral coronal subluxation caused by ACL rupture.

## Methods

### Patients

This retrospective case-control study was conducted in the department of orthopedics at authors’ hospital. After obtaining the approval of the ethical review committee, we searched and collected the data of patients with ACL rupture who completed magnetic resonance imaging (MRI) examination of the knee joint and underwent arthroscopic ACLR in our hospital from January 2015 to January 2020, a total of 59 cases. Inclusion criteria for the study were (1) aged 20 to 60 years old; (2) standard anteroposterior knee radiographs are available; (3) patients with complete ACL rupture and meniscus injury confirmed by arthroscopic examination. Exclusion criteria were (1) inflammatory arthropathy; (2) combined knee fracture; (3) history of surgical procedure on the same knee; (4) multiligament knee injury; (5) Osteoarthritis of the knee, Kellgren-Lawrence grade III or higher; and (6) congenital or secondary malformation deformities. In addition, 30 patients with simple meniscus injury who completed MRI examination of the knee joint and underwent arthroscopy in our hospital during the same period were selected to determine the degree of tibiofemoral joint coronal subluxation as the baseline value, and compared with the patients with ACL rupture combined with meniscus injury. The inclusion criteria of this group of patients were (1) age 20–60 years old; (2) standard anteroposterior knee radiographs are available; (3) medial or lateral meniscus injury confirmed by arthroscopy. Exclusion criteria included (1) inflammatory joint disease; (2) combined with knee ligament injury; (3) combined with knee fracture; (4) Osteoarthritis of the knee, Kellgren-Lawrence grade III or higher; and (5) congenital or secondary malformation deformities.

A total of 11 patients were excluded, including 4 patients with knee fracture, 3 patients with severe osteoarthritis, 3 patients with multiple knee injuries, and 1 patient with inflammatory joint disease. A total of 48 patients met the inclusion and exclusion criteria and were included in the study, including 35 males and 13 females, with a mean age of 37.3 ± 9.9 years and a mean follow-up of 22.3 ± 6.2 months. In the baseline group, there were 30 patients, including 23 males and 7 females, with a mean age of 37.6 ± 10.1 years and a mean follow-up of 21.2 ± 5.8 months.

### Procedures

Single-bundle ACL reconstruction was performed after resecting the native ACL by drilling in the center of the ACL footprints. A quadrupled semitendinosus and gracilis autograft measuring 9 cm in length and 8 to 10 mm in diameter was prepared using an Endobutton (Smith & Nephew Inc., Andover, Massachusetts) and 15-mm loop. The tibial tunnel was created on a plane (tibial tunnel plane) at an angle of 40° from the sagittal plane and should be at an angle of 50° from the tibial axis. The entrance of the lateral femoral bone tunnel is located in the ACL footprint area, and the exit is located above the lateral femoral epicondylar, with a length of about 25-30 mm. Proximal suspension fixation was completed by tying the sutures on a mini plate (Smith & Nephew Inc., Andover, Massachusetts) over the lateral orifice. The tibial side graft was fixed with PEEK interference screw (Smith & Nephew Inc., Andover, Massachusetts) after the femoral notch impingement was excluded. The operation was performed by the same experienced sports medicine surgeon Dr. JZ, he is one of the authors. The meniscus injury was repaired during arthroscopy.

### Radiographic measurement

All patients were taken anteroposterior radiographs in the supine position according to our standard method before and the second day after operation. Care was taken to ensure that each patient lied on the back with their patellae facing upward, to minimize rotational variation among the radiographs. The x-ray beam was centered on the distal pole of the patella and oriented so that the image was aligned parallel to the tibial joint line in the frontal plane. Source to image distance was standardized to 60 cm, and the image included the lower femur and the upper tibia. To assess rotation of the tibia, we used a method based on overlap of the fibula head and tibia previously published by Maderbacher et al. [20]. If the difference between preoperative and postoperative tibial rotation was greater than 5°, the patient was excluded.

**Fig. 1:**
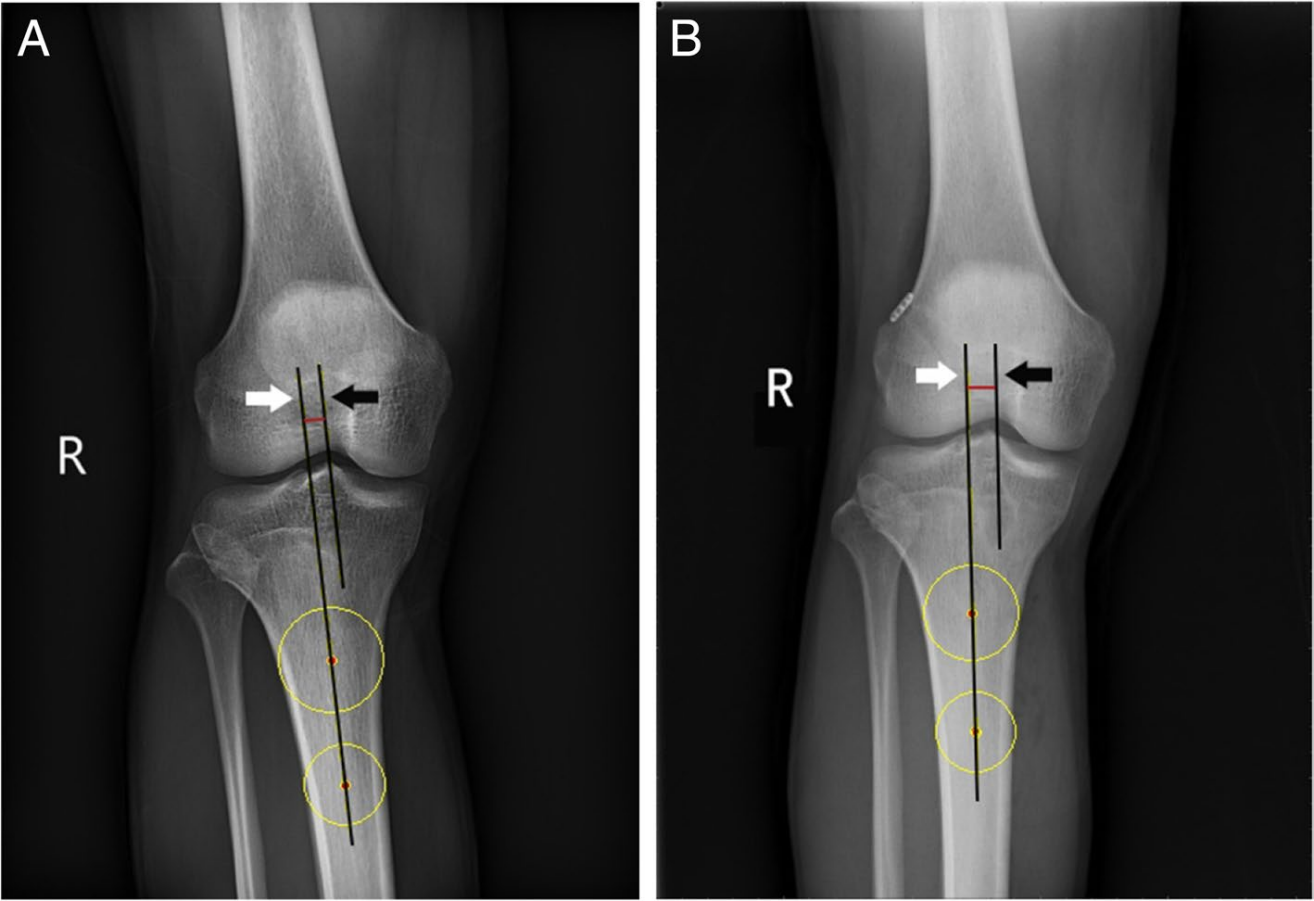
Anteroposterior radiograph of knee. The tibia was displaced medially after anterior cruciate ligament rupture (**A**) and laterally after anterior cruciate ligament reconstruction (**B**). The white arrow points to the line that identifies the long axis of the tibia. The black arrow points to a line parallel to the long axis of the tibia that passes through the most proximal aspect of the femoral intercondylar notch

Tibiofemoral subluxation was previously defined as the perpendicular distance between the long axis of the tibia and a second parallel line originating at the most proximal aspect of the femoral intercondylar notch [21]. To determine the long axis of the tibia, two circles separated by 5 cm were centered on the proximal tibia. The proximal circle is 5 cm from the tibial plateau, and the distal circle is 5 cm from the proximal circle. The line passing through the center of the two circles was considered the long axis of the proximal tibia [22] (Fig. 1). If the line from the apex of the intercondylar notch fell medial to the tibial mechanical axis, the tibiofemoral subluxation was assigned a (+) value (if lateral to the tibial mechanical axis, then a (−) value was assigned). In order to determine whether ACLR can correct the coronal subluxation of tibiofemoral joint after ACL rupture, we measured and compared the coronal subluxation of tibiofemoral joint before and after ACLR. At the same time, we measured the coronal subluxation of tibiofemoral joint in 30 patients with simple meniscus injury. These measurements were performed to provide a baseline value of tibiofemoral subluxation in patients without ACLR.

All radiographic measurements were independently measured by two observers (Li RB and Fu P), and the results were assessed for interobserver reliability. The measurement of radiographs was carried out by the Picture Archiving and Communication System (PACS, Chongqing, China).

### Statistical methods

Quantitative data were expressed as the mean ± standard deviation. After the Kolmogorov-Smirnov test was used to check for normality, 2-tailed paired *t* tests were used to compare the preoperative and postoperative values for tibiofemoral subluxation. After the Kolmogorov-Smirnov test was used to check for normality, independent sample t-test was used to compare the degree of tibiofemoral joint subluxation in the surgery group with that in the baseline group. A t test sample size estimation yielded a group size of 34 patients (alpha, 0.05; power, 0.8; effect size 0.5). Differences were considered significant when *P* < 05. All Statistical analyses were performed using SPSS software (version 25; IBM).

## Results

Demographic data of age, sex, and BMI showed no significant differences between the two groups (Table 1).

**Table 1:** Demographic data

	ACLR group (*n* = 48)	Baseline group (*n* = 30)	*P*
Age (years) ^*a*^	37.3 ± 9.9	37.6 ± 10.1	0.9
Male/ female	35/13	23/7	0.712
BMI (kg/m^2^) ^*a*^	21.7 ± 1.3	21.2 ± 1.5	0.399

*P* values refer to Student t test

*ACLR* anterior cruciate ligament reconstruction

^a^Values are expressed as mean ± standard deviation

The mean tibia lateral subluxation was 5.5 ± 2.1 mm before ACLR, and 7.7 ± 2.6 mm after ACLR, which was significantly different from that before surgery (*P* < 0.001). The lateral tibial subluxation in the baseline group was 7.3 ± 2.1 mm, which was significantly different from that before ACLR (*P* < 0.001), but not significantly different from that after ACLR (*P* = 0.472) (Table 2).

**Table 2:** Coronal plane subluxation and interobserver correlation coefficients

	interobserver correlation coefficients^a^	Coronal plane subluxation, mm^b^	*P*-value
Before ACLR	0.79 (0.76–0.83)	5.5 ± 2.1 (4.1–5.9)	*p*<0.001 ^*^
After ACLR	0.81 (0.77–0.86)	7.7 ± 2.6 (6.0–8.1)	*p* = 0.472 ^**^
Baseline group	0.85 (0.79–0.91)	7.3 ± 2.1 (6.6–8.1)	*p*<0.001 ^***^

*P*-values represent the comparison of coronal plane subluxation in each group

*ACLR* anterior cruciate ligament reconstruction

^a^Values are presented as correlation coefficients (95% CI)

^b^Values are presented as mean ± SD (95% CI)

^*^Before ACLR vs. After ACLR

^**^After ACLR vs. Baseline

^***^Before ACLR vs. Baseline

The interobserver correlation coefficients of tibial subluxation before and after ACLR and baseline group were 0.79 (95%CI, 0.76–0.83), 0.81 (95%CI, 0.77–0.86) and 0.85 (95%CI, 0.79–0.91), respectively.

## Discussion

There are two important findings in this study, one is that the tibia will be subluxated to the medial side after ACL rupture, and the other is that ACLR will make the tibia shift to the lateral side, so as to correct the subluxation of the tibia and restore the tibiofemoral joint to the coronal state before ACL rupture.

The changes in kinematics and the resulting changes in the position of the joint contact points can be explained by the anatomy of ACL. The ACL runs from the superomedial aspect of the lateral femoral condyle in the intercondylar notch to the anterior aspect of the tibial plateau, with an oblique orientation [23]. Because the ACL is oblique, a complete ACL prevents the tibia from moving medially. If the ACL rupture, the tibia will subluxate medially [24]. Previous studies in vivo and in vitro have confirmed that ACL can inhibit medial tibial subluxation [25, 26]. Increased medial translation of the tibia might cause the contact points in the medial and lateral compartments to shift laterally [9]. Increased medial tibial translation could also cause contact between the tibial spine and the medial femoral condyle, elevating the contact stresses in the cartilage. These abnormal contact mechanics might predispose the knee to degenerative arthritis [27].

Understanding the change of tibiofemoral joint relative position and cartilage contact stress after ACL rupture is of great significance for ACLR. Reconstruction of the ACL restores the geometry of the ACL and the pulling effect of the ACL in the coronal plane, allowing the tibia to gain traction and shift medially [10]. In this case, it is reasonable for the knee to return to the coronal plane position it had before the ACL was ruptured.

Although ACLR can reduce the high contact stress of the knee joint with ACL defects, it is still difficult to completely restore the normal mechanical structure of the knee joint, and there is still some residual abnormal contact stress of the knee joint [10, 28]. In ACLR surgery, we usually evaluate the immediate stability of the knee and the tension of the ACL after ACLR according to the distance of tibial forward displacement (e.g., anterior drawer test) [29]. However, it is difficult to find an effective method to evaluate the lateral stability, that is, the movement of the coronal plane of the tibia. Previous studies have shown that the abnormal contact stress of the knee joint after ACLR is not only related to the anteroposterior displacement of the contact point of the knee joint, but also has an important relationship with the coronal plane displacement. Therefore, intraoperative measurement of the degree of tibiofemoral joint coronal subluxation is also of great significance for evaluating the effect of ACLR. In this study, we found that the tibia would shift laterally after ACLR. If we can get the distance of tibial coronal plane displacement by C-arm radiographs during ACLR surgery, it will be very meaningful to evaluate the effect of ACLR.

This study has several limitations: (1) a prospective study including radiographs of the limb before and after ACL injury would be superior to our retrospective study. There is no way to obtain routine knee radiographs of patients before ACL injury. (2) Since the number of patients with simple ACL rupture was very small, the patients included in our study were patients with ACL rupture combined with meniscus injury. However, we selected 30 patients with simple meniscus injury and measured baseline data for tibiofemoral joint coronal subluxation. The design of baseline control group was able to offset the confounding factor of meniscus injury. (3) The meniscus injury types were not grouped in this study. It is not clear whether different types of meniscus injuries have different effects on tibiofemoral joint coronal subluxation. (4) measurement of subluxation on radiographs is an imperfect way of evaluating the structural pathology within the knee and more accurate techniques for assessing subluxation are warranted. (5) The sample size of this study was smaller than the estimated sample size, which affected the effect size of the test. In subsequent studies, we will increase the sample size, improve the effect size of the test, and analyze the differences between genders.

## Conclusions

This study showed that the tibia can be subluxated medially after ACL rupture. ACLR can correct the medial subluxation of tibia. This finding is helpful in the diagnosis of ACL rupture, and can be used to assess the imaging status of the tibiofemoral joint on the coronal plane during or after ACLR.

## Data Availability

The datasets used and/or analyzed during the current study are available from the corresponding author on reasonable request.

## Abbreviations

ACL: Anterior cruciate ligament
ACLR: Anterior cruciate ligament reconstruction
DR: Digital X-ray imaging systems

## References

[1] Luc B, Gribble PA, Pietrosimone BG (2014). Osteoarthritis prevalence following anterior cruciate ligament reconstruction: a systematic review and numbers-needed-to-treat analysis. J Athl Train.

[2] Zampeli F, Ntoulia A, Giotis D, Stavros R, Mitsionis G, Pappas E, Georgoulis AD, Georgoulis AD (2018). The PCL index is correlated with the control of rotational kinematics that is achieved after anatomic anterior cruciate ligament reconstruction. Knee Surg Sports Traumatol Arthrosc.

[3] Murawski CD, Eck CF, Irrgang JJ, Tashman S, Fu FH (2014). Operative treatment of primary anterior cruciate ligament rupture in adults. J Bone Joint Surg Am.

[4] Korpershoek JV, de Windt TS, Vonk LA, Krych AJ, Saris DBF. Does anterior cruciate ligament reconstruction protect the Meniscus and its repair? A Systematic Review. Orthop J Sports Med. 2020;8(7):2325967120933895. 10.1177/2325967120933895 PMC7388123 32782901

[5] Ajuied A, Wong F, Smith C, Norris M, Earnshaw P, Back D, Davies A (2014). Anterior cruciate ligament injury and radiologic progression of knee osteoarthritis: a systematic review and meta-analysis. Am J Sports Med.

[6] Moksnes H, Risberg MA (2009). Performance-based functional evaluation of non-operative and operative treatment after anterior cruciate ligament injury. Scand J Med Sci Sports.

[7] Defrate LE, Papannagari R, Gill TJ, Moses JM, Pathare NP, Li G (2006). The 6 degrees of freedom kinematics of the knee after anterior cruciate ligament deficiency: an in vivo imaging analysis. Am J Sports Med.

[8] Kopf S, Musahl V, Bignozzi S, Irrgang JJ, Zaffagnini S, Fu FH (2014). In vivo kinematic evaluation of anatomic double-bundle anterior cruciate ligament reconstruction. Am J Sports Med.

[9] Khamaisy S, Zuiderbaan HA, Thein R, Gladnick BP, Pearle AD (2016). Coronal tibiofemoral subluxation in knee osteoarthritis. Skelet Radiol.

[10] Imhauser C, Mauro C, Choi D, Rosenberg E, Mathew S, Nguyen J (2013). Abnormal tibiofemoral contact stress and its association with altered kinematics after center-center anterior cruciate ligament reconstruction: an in vitro study. Am J Sports Med.

[11] Fairclough JA, Graham G, Dent CM (1990). Radiological sign of chronic anterior cruciate ligament deficiency. Injury..

[12] Segal NA, Anderson DD, Iyer KS, Baker J, Torner JC, Lynch JA (2009). Baseline articular contact stress levels predict incident symptomatic knee osteoarthritis development in the MOST cohort. J Orthop Res.

[13] LaPrade RF, Moulton SG, Nitri M, Mueller W, Engebretsen L (2015). Clinically relevant anatomy and what anatomic reconstruction means. Knee Surg Sports Traumatol Arthrosc.

[14] Zampeli F, Ntoulia A, Giotis D, Tsiaras VA, Argyropoulou M, Pappas E (2012). Correlation between anterior cruciate ligament graft obliquity and tibial rotation during dynamic pivoting activities in patients with anatomic anterior cruciate ligament reconstruction: an in vivo examination. Arthroscopy..

[15] Herbort M, Domnick C, Raschke MJ, Lenschow S, Förster T, Petersen W, Zantop T (2016). Comparison of knee kinematics after single-bundle anterior cruciate ligament reconstruction via the medial portal technique with a central femoral tunnel and an eccentric femoral tunnel and after anatomic double-bundle reconstruction: a human cadaveric study. Am J Sports Med.

[16] Mohtadi N, Chan D, Barber R, Oddone PE (2015). A randomized clinical trial comparing patellar tendon, hamstring tendon, and double-bundle ACL reconstructions: patient-reported and clinical outcomes at a minimal 2-year follow-up. Clin J Sport Med.

[17] Porter MD, Shadbolt B (2014). "Anatomic" single-bundle anterior cruciate ligament reconstruction reduces both anterior translation and internal rotation during the pivot shift. Am J Sports Med.

[18] Ristanis S, Stergiou N, Siarava E, Ntoulia A, Mitsionis G, Georgoulis AD (2009). Effect of femoral tunnel placement for reconstruction of the anterior cruciate ligament on tibial rotation. J Bone Joint Surg Am.

[19] Zampeli F, Terzidis I, Espregueira-Mendes J, Georgoulis JD, Bernard M, Pappas E, Georgoulis AD (2018). Restoring tibiofemoral alignment during ACL reconstruction results in better knee biomechanics. Knee Surg Sports Traumatol Arthrosc.

[20] Maderbacher G, Schaumburger J, Baier C, Zeman F, Springorum HR, Springorum HR (2014). Predicting knee rotation by the projection overlap of the proximal fibula and tibia in long-leg radiographs. Knee Surg Sports Traumatol Arthrosc.

[21] Nam D, Khamaisy S, Gladnick BP, Paul S, Pearle AD (2013). Is tibiofemoral subluxation correctable in unicompartmental knee arthroplasty?. J Arthroplast.

[22] Li R, Yuan X, Fang Z, Liu Y, Chen X, Zhang J (2020). A decreased ratio of height of lateral femoral condyle to anteroposterior diameter is a risk factor for anterior cruciate ligament rupture. BMC Musculoskelet Disord.

[23] Li G, Defrate LE, Rubash HE, Gill TJ (2005). In vivo kinematics of the ACL during weight-bearing knee flexion. Clin Biomech (Bristol, Avon).

[24] Li G, Papannagari R, DeFrate LE, Yoo JD, Park SE, Gill TJ (2007). The effects of ACL deficiency on mediolateral translation and varus-valgus rotation. Acta Orthop.

[25] Li G, Park SE, DeFrate LE, Schutzer ME, Ji L, Gill TJ, Rubash HE (2005). The cartilage thickness distribution in the tibiofemoral joint and its correlation with cartilage-to-cartilage contact. Clin Biomech (Bristol, Avon).

[26] Li G, Papannagari R, DeFrate LE, Yoo JD, Park SE, Gill TJ (2006). Comparison of the ACL and ACL graft forces before and after ACL reconstruction: an in-vitro robotic investigation. Acta Orthop.

[27] Li G, Moses JM, Papannagari R, Pathare NP, DeFrate LE, Gill TJ (2006). Anterior cruciate ligament deficiency alters the in vivo motion of the tibiofemoral cartilage contact points in both the anteroposterior and mediolateral directions. J Bone Joint Surg Am.

[28] Noyes FR, Huser LE, Levy MS (2018). The effect of an ACL reconstruction in controlling rotational knee stability in knees with intact and physiologic laxity of secondary restraints as defined by Tibiofemoral compartment translations and graft forces. J Bone Joint Surg Am.

[29] Yamamoto Y, Tsuda E, Maeda S, Naraoka T, Kimura Y, Chiba D, Ishibashi Y (2018). Greater laxity in the anterior cruciate ligament-injured knee carries a higher risk of Postreconstruction pivot shift: intraoperative measurements with a navigation system. Am J Sports Med.

